# Ewing sarcoma with very late metastasis in the skull: a case report

**DOI:** 10.1186/s13256-022-03656-5

**Published:** 2022-11-15

**Authors:** Ryota Hagihara, Hidetaka Arishima, Takahiro Yamauchi, Satoshi Kawajiri, Tomomi Ito, Mana Fukushima, Kenichiro Kikuta

**Affiliations:** 1 Division of Medicine, Department of Neurosurgery, Faculty of Medical Sciences, University of Fukui, 23-3 Matsuokashimoaizuki, Eiheiji-Cho, Yoshida-Gun, Fukui, 910-1193 Japan; 2 Division of Diagnostic Pathology/Surgical Pathology, University of Fukui Hospital, 23-3 Matsuokashimoaizuki, Eiheiji-Cho, Yoshida-Gun, Fukui, 910-1193 Japan; 3 Department of Tumor Pathology, Faculty of Medical Sciences, University of Fukui, 23-3 Matsuokashimoaizuki, Eiheiji-Cho, Yoshida-Gun, Fukui, 910-1193 Japan

**Keywords:** Brain neoplasms, Ewing sarcoma, Late metastasis, Case report

## Abstract

**Background:**

Ewing sarcoma is a malignant bone tumor; however, its prognosis has improved since the development of modern chemotherapy. Although Ewing sarcoma outcomes have improved, issues related to late complications, secondary malignant neoplasms, and late recurrence or metastasis have emerged.

**Case presentation:**

We report a case of Ewing sarcoma that recurred in the occipital bone 21 years after primary tumor treatment. A 45-year-old Japanese woman with a history of Ewing sarcoma 21 years prior, was referred to our hospital due to a severe headache. A tumor was detected in the left occipital bone, and the biopsy revealed Ewing sarcoma. Metastasis was suspected because the patient had been treated for Ewing sarcoma of the left clavicle 21 years prior. There have been several cases of local recurrence or metastasis, occurring 15–20 years after the onset of the initial disease. To our knowledge, very late metastasis of Ewing sarcoma in the skull has not been reported.

**Conclusion:**

We report a rare case of very late metastasis of Ewing sarcoma in the skull with a review of the literature. Delayed metastasis secondary to Ewing sarcoma can occur in the lung, which is the most common site for metastasis, as well as other regions of the body, such as the cranium.

**Supplementary Information:**

The online version contains supplementary material available at https://doi.org/10.1186/s13256-022-03656-5.

## Background

Ewing sarcoma (ES) is a primary malignant bone tumor that occurs in approximately 30–40 patients annually in Japan [1]. The combination of preoperative and postoperative chemotherapy has improved treatment outcomes [2]. However, late complications and second malignant neoplasms (SMNs) remain a problem, and these require a long follow-up period after treatment [1, 2]. Although recurrence typically occurs within 5 years of treatment [3, 4], there have been several reports on local recurrence or metastasis after 15 years or more [5–7]. However, the very late metastasis of ES in the skull has not been reported. This report documents a case of ES recurrence in the skull 21 years after the onset of the initial disease, and reviews the related literature.

## Case presentation

**Fig. 1:**
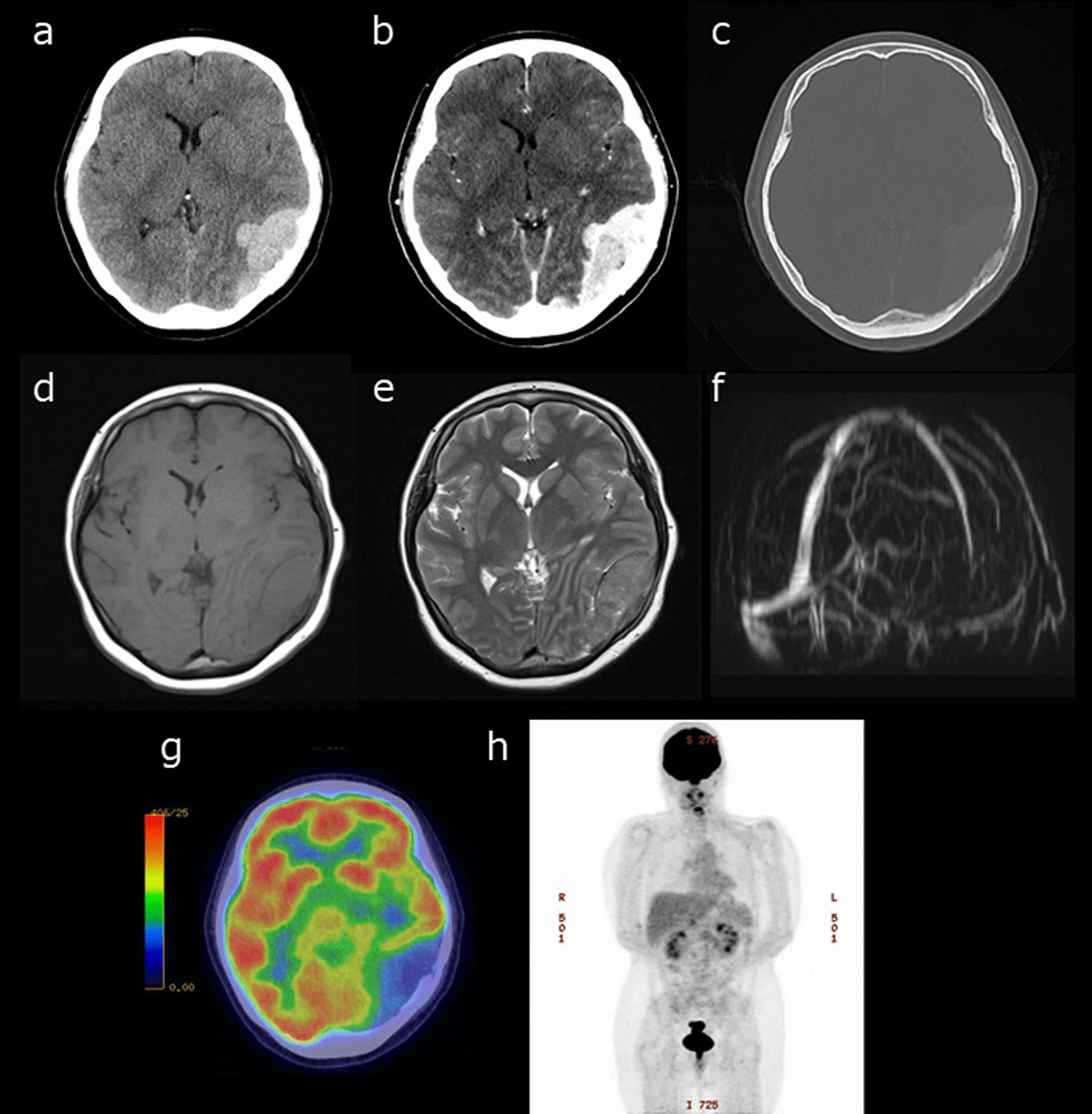
Precontrast head computed tomography (CT) shows a high-density lesion bordering the left occipital bone (**a**), and the contrast-enhanced head CT shows a contrast enhancement effect (**b**). Osteolytic changes are also observed in the left occipital bone (**c**). On the head magnetic resonance imaging (MRI), the lesion exhibits isodense signal intensity on T1-weighted imaging (**d**) and T2-weighted imaging (**e**). Magnetic resonance venography shows no signal in the left transverse sinus (**f**). ^18^F-fluorodeoxyglucose (^18^F-FDG) positron emission tomography-CT reveals low.^18^F-FDG uptake in the tumor (**g**) and no abnormally high uptake throughout the body (**h**)

A 45-year-old Japanese woman was referred to our department due to a severe headache. Twenty-one years prior, she was diagnosed with left clavicle ES and underwent surgical tumor excision. Preoperative and postoperative chemotherapy, consisting of vincristine, cyclophosphamide, doxorubicin, ifosfamide and etoposide were respectively administered. The patient also had a history of left breast cancer, treated with pectoral muscle-sparing mastectomy. Preoperative and postoperative chemotherapy, consisting of docetaxel, pertuzumab, trastuzumab, fluorouracil, epirubicin, and cyclophosphamide, were respectively administered 2 years prior. On admission, the patient presented with a headache, but no neurological findings were noted. Contrast-enhanced computed tomography (CT) of the brain showed a tumor extending superiorly and inferiorly from the cerebellar tent, along the left occipital bone with a contrast enhancement effect (Fig. 1a, b). Osteolytic changes were observed in the left occipital bone (Fig. 1c). Magnetic resonance imaging (MRI) of the brain revealed the tumor, exhibiting an isodense signal intensity on T1-weighted (Fig. 1d) and T2-weighted imaging (Fig. 1e). No signal was noted in the left transverse sinus on magnetic resonance venography. This suggested occlusion due to tumor invasion (Fig. 1f). The tumor extended to the left mastoid air cells. On ^18^F-fluorodeoxyglucose-positron emission tomography (^18^F-FDG PET), the tumor exhibited a low uptake (Fig. 1g). No high uptake regions were observed throughout the entire body (Fig. 1h).

**Fig. 2:**
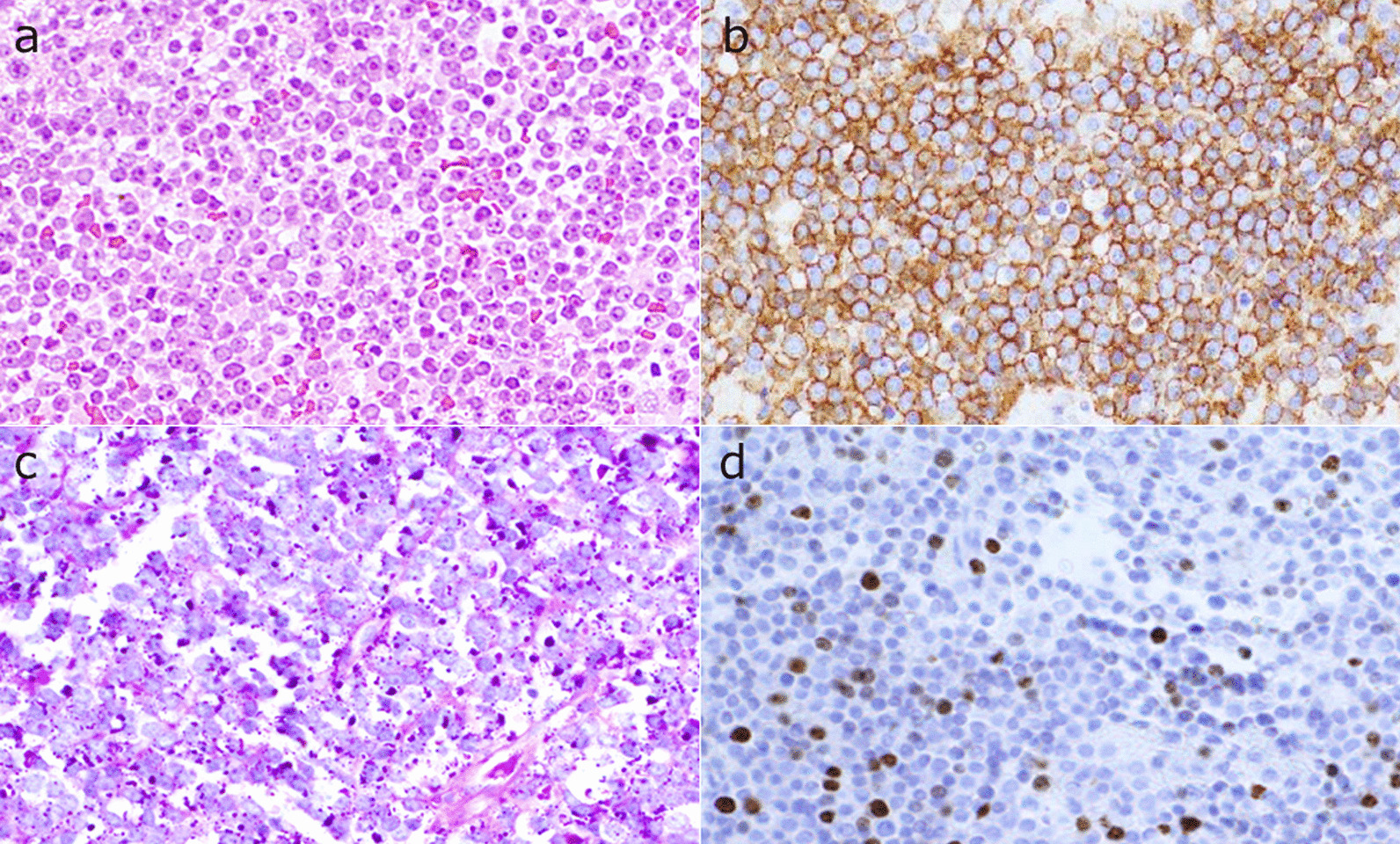
Hematoxylin–eosin staining shows small round cells with little cytoplasm (**a** ×200). Immunohistochemical staining shows a membranous expression of CD99 (**b** ×200). Periodic acid-Schiff staining detected small positive granules (**c** ×200). Ki-67 staining shows that the MIB-1 index is about 30% (**d** × 200)

Following admission, a small craniotomy and biopsy were performed to confirm the pathological diagnosis. Pathological examination showed small round cells with scanty cytoplasm on hematoxylin–eosin staining (Fig. 2a). CD99 immunohistochemistry showed strong membranous staining among the tumor cells (Fig. 2b). Periodic acid-Schiff detected small positive granules in the cytoplasm (Fig. 2c). Therefore, the patient was diagnosed with bone metastasis of ES. A craniotomy and subtotal tumor excision were performed the following day. After the subtotal removal of metastatic tumor, her headache improved.

**Fig. 3:**
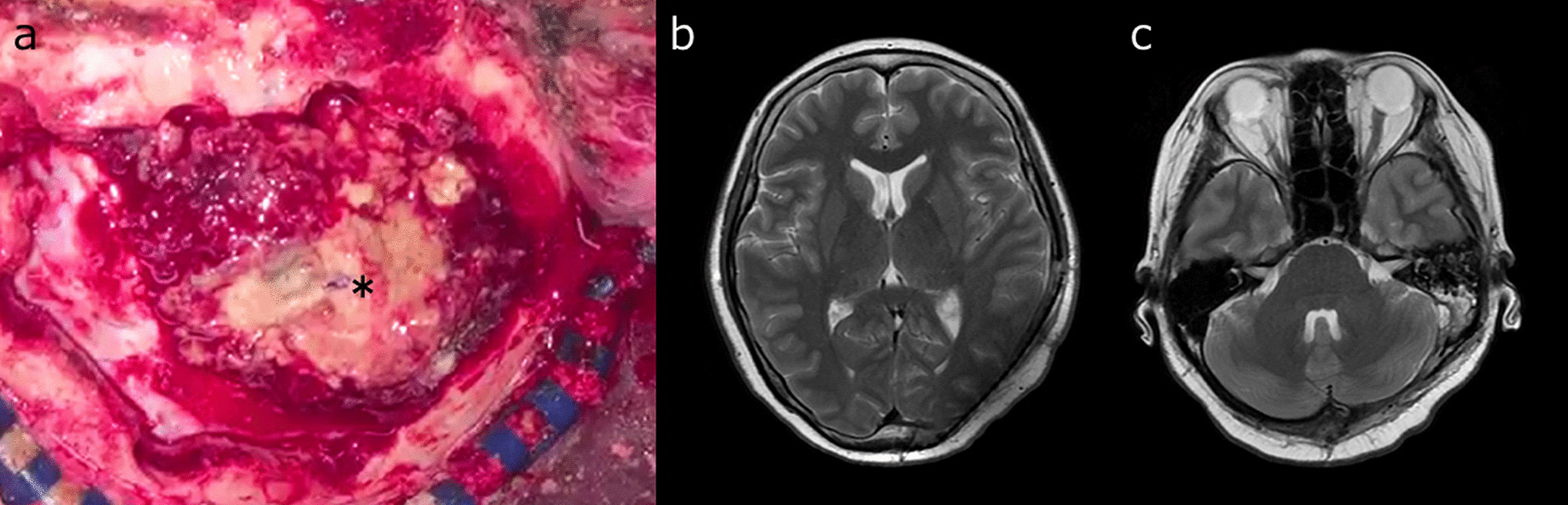
Craniotomy was performed across the transverse sinus. A yellowish-white tumor (asterisk) was observed in the epidural space (**a**). Head MRI after intensity-modulated radiation therapy shows the residual tumor in the left mastoid air cells and sigmoid sinus; however, it is smaller than that before radiotherapy (**b**, **c**)

During the craniotomy, a yellowish-white neoplastic lesion was observed in the epidural space across the transverse sinus (Fig. 3a). A portion of the tumor infiltrated the dura and extended to the surface of the brain. The tumor was removed as completely as possible, and the left transverse sinus was resected. However, the tumors in the left mastoid air cells and left sigmoid sinus could not be excised. Postoperatively, intensity-modulated radiation therapy (IMRT) at 61.2 Gy was administered in 34 fractions. Head MRI after IMRT showed a small residual tumor (Fig. 3b, c). After 2 months of radiotherapy, the patient was discharged without headache, and there was no noted deterioration of her status. Genetic examination (OncoGuide NCC OncoPanel System) indicated *BRCA1*–E1148fs* and *EWSR1*–*FLI1* fusion, and chemotherapy with pembrolizumab (platinum) and olaparib (poly adenosine diphosphate-ribose polymerase inhibitor) was considered. However, chemotherapy was not performed because of the low grade of evidence for both drugs. At present, approximately 9 months after surgery, MRI shows no local recurrence.

## Discussion

ES is a malignant tumor that arises from bones or soft tissues, and is characterized by primitive small and round cells [1, 8]. Pathological and molecular similarities have been detected among primary ES, extraosseous ES, peripheral primitive neuroectodermal tumors, and Askin tumors. Collectively, these comprise the ES family of tumors (ESFT). Tumors classified under the ESFT exhibit similar types of chromosomal translocations, such as t(11;22)(q24;q12) [6]. The standard treatment for ES is a multimodal therapy, which includes surgery, systemic chemotherapy, and radiotherapy. The 5-year event-free survival rate has increased to 60–70% due to the advancements in the treatment modalities [1, 2]. The survival rate of ES has also increased. However, SMNs or late recurrences, that occur more than 10 years after the primary treatment, have been reported [9]. While more than 90% of recurrences occur within the first 5 years [3, 4], no standard treatment strategy for recurrent ES has been established [1]. Furthermore, the 5-year survival rate remains below 20% in patients with recurrent or metastatic tumors [8]. A systemic workup is essential for proper prognostication [9].

We encountered a case of ES, that recurred in the cranium, 21 years after the onset of the initial disease. Four cases with the development of local recurrence or metastasis more than 10 years after treatment had previously been reported [5–7]. However, there have been no reports of cranial metastasis, occurring 20 years after the initial treatment (Additional file 1: Table S1). Based on previous reports available at the time of this study, the longest interval between the initial ES diagnosis and metastasis is 29 years. The patient was diagnosed with ES of the right femur and underwent extensive resection, preoperative and postoperative chemotherapy, and postoperative radiotherapy. However, a late lung metastasis was found 29 years later [7]. Anti-angiogenic signals were reportedly integral in maintaining the dormancy of osteosarcoma cells [10]. The mechanisms behind tumor dormancy in sarcomas have been thoroughly investigated. However, there have been no studies describing dormant ES [10]. Furthermore, the fusion protein EWSR1–*FLI1*, produced by the chromosomal translocation, functions as a transcription factor in ES. Although it is considered an ideal target for treating ES, most studies targeting this signaling are still in the initial stages of development [8]. However, it has been suggested that the mismatch repair (MMR) pathway, which is highly activated during the G1/S-phase, may contribute to ES proliferation, invasion, and migration [11]. The MMR pathway including *MSH2*, *MSH4*, *RFC2*, and *RPA2*, is reported to be closely correlated with poor prognosis of ES patients. It has been confirmed that knockdown of EWSR1–*FLI1* impairs ES tumorigenesis in vivo, suggesting that the expression of these four genes may be partially regulated by EWSR1–*FLI1* fusion [11].

ES frequently metastasizes to the lung, while cranial metastasis is rare, occurring in only 1% of ES cases [1, 12, 13]. The present case emphasizes the importance of a continuous follow-up schedule and thorough systemic evaluation. This includes evaluating the brain for local recurrence and metastasis, even in patients who have been treated more than 20 years prior.

The sensitivity and specificity of ^18^F-FDG PET for ES are reportedly 73% and 83%, respectively [14]. In the present case, ^18^F-FDG PET yielded a negative result for ES. This was likely caused by the altered glucose metabolism induced by ES metastasis [15]. Although whole-body MRI, ^18^F-FDG PET-CT, and 99mTc-MDP skeletal scintigraphy are usually used to detect osseous metastases of ES, there is no literature demonstrating the difference in the sensitivities and specificities of these imaging modalities [9, 15].

Despite the need for a long-term evaluation, the laboratory workup modalities remain incomplete. Physicians should acknowledge that ES recurrence or metastasis develops late in the course of treatment; thus, a continuous follow-up schedule is necessary. There is no complete investigation tool. Late recurrence or metastasis of ES in any part of the body is possible; therefore, we should continue careful follow-up over decades.

## Conclusion

We report a case of ES that recurred in the occipital bone 21 years after the initial diagnosis. Although it is known that ES frequently metastasizes to the lungs and rarely to the central nervous system and skull, we should be aware that ES can metastasize not only to the lungs, but also to the whole body, including the skull.

## Supplementary Information

**Additional file 1: Table S1.** Summary of late local recurrence or metastasis of Ewing sarcoma family of tumors [see Addtional file 1]. *LR* local recurrence; *M* metastases; *ANED* alive no evidence of disease; *AWED* alive with evidence of disease.

## Data Availability

Data sharing is not applicable to this article as no datasets were generated or analyzed during the current study.

## Abbreviations

ES: Ewing sarcoma
SMNs: Second malignant neoplasms
CT: Computed tomography
MMR: Mismatch repair
MRI: Magnetic resonance imaging
^18^F-FDG PET: ^18^F-fluorodeoxyglucose-positron emission tomography
IMRT: Intensity-modulated radiation therapy
ESFT: Ewing sarcoma family of tumors
